# Plastic anisotropy and dislocation trajectory in BCC metals

**DOI:** 10.1038/ncomms11695

**Published:** 2016-05-25

**Authors:** Lucile Dezerald, David Rodney, Emmanuel Clouet, Lisa Ventelon, François Willaime

**Affiliations:** 1 DEN-Service de Recherches de Métallurgie Physique, CEA, Université Paris-Saclay, F-91191 Gif-sur-Yvette, France; 2 Institut Jean Lamour, Université de Lorraine - CNRS, F-54011 Nancy, France; 3 Institut Lumière Matière, Université Lyon 1 - CNRS, F-69622 Villeurbanne, France; 4 DEN-Département des Matériaux pour le Nucléaire, CEA, Université Paris-Saclay, F-91191 Gif-sur-Yvette, France

## Abstract

Plasticity in body-centred cubic (BCC) metals at low temperatures is atypical, marked in particular by an anisotropic elastic limit in clear violation of the famous Schmid law applicable to most other metals. This effect is known to originate from the behaviour of the screw dislocations; however, the underlying physics has so far remained insufficiently understood to predict plastic anisotropy without adjustable parameters. Here we show that deviations from the Schmid law can be quantified from the deviations of the screw dislocation trajectory away from a straight path between equilibrium configurations, a consequence of the asymmetrical and metal-dependent potential energy landscape of the dislocation. We propose a modified parameter-free Schmid law, based on a projection of the applied stress on the curved trajectory, which compares well with experimental variations and first-principles calculations of the dislocation Peierls stress as a function of crystal orientation.

## Abstract (web summary)

Low-temperature plasticity in BCC metals is atypical, marked by an anisotropic elastic limit in violation of the Schmid law. Here, the authors show that these deviations can be quantified from subatomic deviations of the screw dislocation trajectory away from its average glide plane.

## Graphical abstract

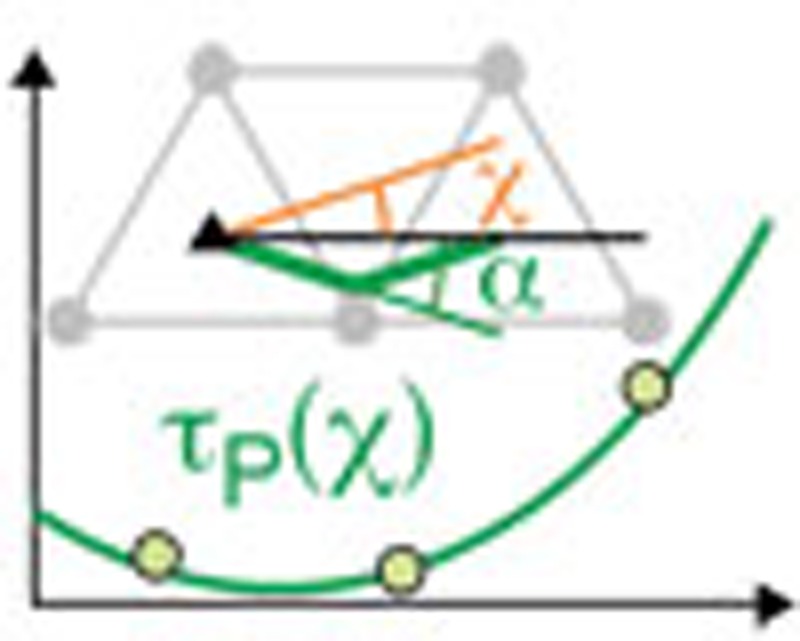

The crystalline structure of metals strongly affects their plasticity. In face-centred cubic and hexagonal close-packed metals with basal slip, plastic flow sets in when the resolved shear stress (RSS, the component of the applied stress tensor that produces a shear stress along the slip direction and parallel to the slip plane) reaches a critical value. In this yield criterion, famously known as the Schmid law^1^, the critical RSS is a structural parameter dependent on the thermomechanical history of the metal, but not on the sense nor on the direction of the applied stress.

Body-centred cubic (BCC) metals are known to disobey the Schmid law, in particular because the critical RSS depends on the orientation and sense of the applied stress^2^,^3^,^4^. BCC metals slip along dense 〈111〉 directions, but the potential slip planes vary depending on the element, the temperature and orientation of the maximum RSS plane^3^,^5^. Plastic anisotropy in these metals was initially interpreted as resulting from slip on {112} planes^2^,^6^. The reason is that these planes are not symmetrical with respect to shear along 〈111〉, with a direction leading to twinning (T), softer than the opposite, antitwinning (AT), direction. However, this hypothesis does not hold, as the T/AT asymmetry remains at low temperatures in α-Fe and in metals from group VI (Mo and W), which slip along {110} planes that are symmetrical with respect to 〈111〉 shear^3^,^7^. Moreover, screw dislocations with 1/2〈111〉 Burgers vectors, which are known to be the source of the plastic anisotropy^4^,^8^, have been shown via atomistic calculations to disobey the Schmid law even when they glide on {110} planes^9^,^10^,^11^. Another feature, perhaps less commonly known, is that deviations from the Schmid law are metal dependent^12^.

To describe non-Schmid effects, a phenomenological yield criterion was proposed as a linear combination of the stresses that affect plasticity^13^,^14^. The stresses were identified through atomistic simulations^9^,^14^,^15^ and the resulting generalized yield criterion was shown to reproduce accurately atomistically calculated anisotropic Peierls stresses, that is, the critical RSS to set an isolated screw dislocation in motion in the absence of thermal activation^10^,^14^,^16^,^17^. However, there has been so far no physical explanation of the dependence of the yield criterion on these various stress components nor of the variation among metals of the parameters entering the law, reflecting our lack of understanding of the T/AT asymmetry.

We show here using density functional theory (DFT) calculations that the link between the T/AT asymmetry and screw dislocations lies in the dislocation trajectory that deviates from the average {110} slip plane. Applying the Schmid law to the actual dislocation trajectory instead of the average {110} glide plane allows the physical interpretation of the phenomenological generalized yield criterion and leads to predictions of T/AT asymmetries that are in qualitative agreement with experiments and in quantitative agreement with DFT calculations of the Peierls stress.

## Results

### T/AT asymmetry

**Figure 1:**
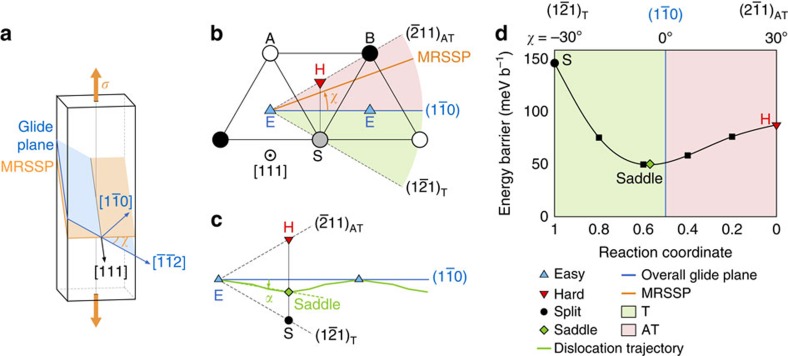
BCC metals plasticity and crystallography. (**a**) Schematic representation of the (1
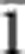
0) glide plane and maximum RSS plane (MRSSP) during a tensile test. (**b**) Projection of the BCC lattice in the (111) plane showing the high-symmetry dislocation positions (E, easy core; H, hard core; S, split core). The angle *χ* between the (1
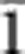
0) plane and the maximum RSS plane is positive in the AT region (red area) and negative in the T region (green area). (**c**) Example of dislocation trajectory (green line) calculated with DFT in Mo and definition of the deviation angle *α*. The green diamond shows the saddle point position. (**d**) Energy profile along the hard-split line (H-S segment in **b**,**c**) calculated with DFT in Mo.

To examine plastic anisotropy, we use first-principles DFT calculations to determine the energetics and trajectories of **b**=1/2[111] screw dislocations gliding between neighbouring equilibrium configurations. We consider the 0 K Peierls stress limit where dislocations glide rigidly, allowing to simulate straight periodic dislocation lines. The calculations are performed both in the absence and in the presence of a shear stress applied in the maximum RSS plane. This plane belongs to the [111] zone but may differ from the (1
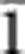
0) plane, as encountered in tensile tests (Fig. 1a). We considered the following BCC transition metals: V, Nb and Ta from group V; Mo and W from group VI; and Fe. The energy barriers against glide, as a function of the applied stress and orientation of the maximum RSS plane, are obtained by a constrained minimization and the dislocation positions defining the corresponding trajectory are deduced from the stress variations calculated along the paths (for details, see Methods).

Figure 1b shows the BCC structure projected in the (111) plane, along with the high-symmetry positions of the 1/2〈111〉 screw dislocation. The easy core configuration is centred on upward triangles formed by three [111] atomic columns and corresponds to the minimum-energy stable position of the dislocation. The hard core is an unstable position centred on downward triangles, where the three [111] columns are at the same altitude. We used the split core configuration to model the dislocation in the vicinity of a [111] atomic column. It corresponds to altitude shifts of ±*b*/6 for the two opposing columns (columns A and B for a split core near column S in the ABS triangle)^18^,^19^. The split core is unstable in DFT calculations^19^,^20^ but metastable with some interatomic potentials^21^,^22^,^23^. The T/AT asymmetry arises when a simple shear stress is applied along the [111] direction with a maximum RSS plane that is not the central (1
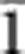
0) plane^4^. The orientation of the maximum RSS plane is commonly referred to by its angle *χ* relative to the (1
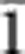
0) plane, as illustrated in Fig. 1a,b. {112} planes are not symmetrical when sheared along the [111] direction. For positive stresses, when *χ*=+30°, the (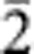
11) plane is sheared in the AT direction, whereas when *χ*=−30°, the (1
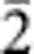
1) plane is sheared in the T direction. The region with *χ*>0 (respectively *χ*<0) is therefore referred to as antiwinning (respectively twinning). Interestingly, the T/AT asymmetry is also visible in the topology of the (111) plane (see Fig. 1b), as the (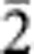
11) plane in the AT region contains the hard core, whereas the (1
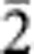
1) plane in the T region contains a [111] atomic column. Therefore, a shear stress applied along a maximum RSS plane with *χ*>0 pushes the dislocation towards the hard core, whereas when *χ*<0 the dislocation is pushed towards the split core.

### Dislocation trajectory

The Peierls barriers between two neighbouring easy cores were calculated in all BCC metals and the corresponding dislocation trajectories were determined from the stress variations observed along the paths (see Methods). Extracting these trajectories from the atomistic simulations through a fit of the atomic displacements rather than using the stress variation leads qualitatively to the same trajectories (see Supplementary Fig. 1). An example of dislocation trajectory in the absence of an applied stress is shown in Fig. 1c. As expected from previous studies^19^,^20^,^24^,^25^,^26^,^27^, the trajectory is not straight and the maximum deviation is reached halfway across the path when the dislocation is in the saddle state of maximum energy between the initial and final configurations. At this point, the dislocation trajectory crosses the line that connects the hard core to the split core configurations, hereafter called the hard-split line. As the trajectories are close to piecewise linear, we will characterize the deviation of a path from the straight line by the angle *α* between the first section of the trajectory and the horizontal (1
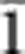
0) plane (see Fig. 1c).

The *α* angles computed in the absence of applied stress are given in Table 1. They are systematically negative, reflecting that the deviation always points towards the [111] atomic column located in the T region. This can be understood from the dislocation two-dimensional (2D) Peierls potential (the energy landscape that describes the interaction between the dislocation and the crystal lattice)^20^,^24^,^25^,^26^,^27^,^28^. The energy along the hard-split line calculated with DFT is illustrated in Mo in Fig. 1d. This energy profile is strongly asymmetric with respect to the central (1
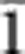
0) plane and the local minimum corresponding to the saddle point of the 2D Peierls potential is shifted towards the split core in the T region. As the gliding dislocation tends to follow the path of minimum energy on the Peierls potential, the shift of the saddle point position relative to the (1
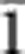
0) plane explains the negative *α* angles in Table 1.

**Figure 2:**
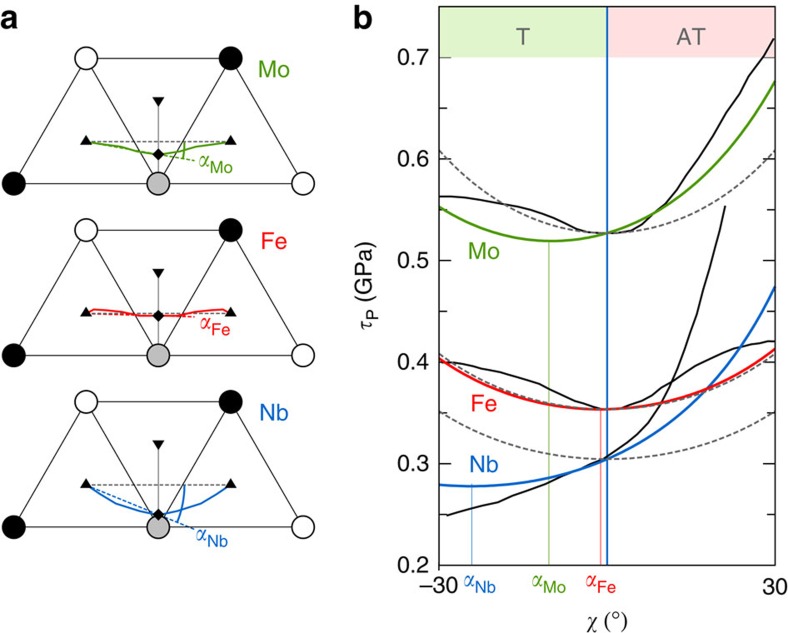
Dislocation trajectory and Schmid law deviation in BCC metals. (**a**) Dislocation trajectory between easy core configurations obtained by DFT in Nb, Mo and Fe, with the corresponding deviation angle *α*. (**b**) Peierls stress variations with crystal orientations predicted by the modified Schmid law of equation (2) (coloured lines) and compared with the experimental data at 4.2 K (ref. 12) (black lines). Schmid law is indicated in dashed grey lines.

We also note that the magnitude of the deviation of the dislocation trajectory is strongly metal dependent. As illustrated in Fig. 2a, the deviation is largest in Nb, where the dislocation passes close to the [111] column, intermediate in Mo and minimum in *α*-Fe, where the path is almost straight. This is a direct consequence of the asymmetry of the hard-split line, which is itself strongly metal dependent^20^. We reported in Table 1 the energy ratios between the split and hard cores, *E*_S_/*E*_H_, taking as a reference the easy core. We see that, with the exception of V, the split core energy relative to the hard core energy controls the saddle point position, that is, the larger the *E*_S_/*E*_H_ ratio, the smaller the deviation angle. In particular, Fe has the largest ratio and the lowest *α*-value, whereas Nb has an *E*_S_/*E*_H_ ratio close to 1 and the largest deviation angle. The reason why the energy ratios are metal dependent remains to be understood; however, further explanations require investigations that are out of the scope of the present publication.

### Modified Schmid law

The Schmid law assumes that the critical RSS is controlled by the shear stress projected on the glide plane. If a shear stress τ is applied in the maximum RSS plane, the RSS in the (1
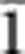
0) glide plane is 
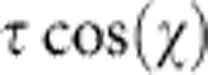
. Applying the Schmid law, the screw dislocation Peierls stress should then vary with the maximum RSS plane orientation as:


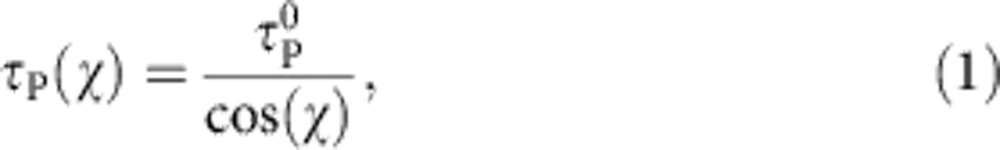


which is symmetrical with respect to the central (1
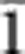
0) plane where *χ*=0°. However, we have shown above that in BCC metals, the dislocation trajectory is (1
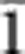
0) only on average. To obtain the Peierls stress, one should therefore project the RSS not on the average (1
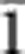
0) plane but rather on the actual trajectory. As the trajectories are close to piecewise linear with the unstable position of the dislocation under stress in the first half of the path, we propose to project the RSS on the plane inclined by an angle *α* with respect to the horizontal (1
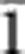
0) plane. We thus obtain a modified Schmid law, expressed as:


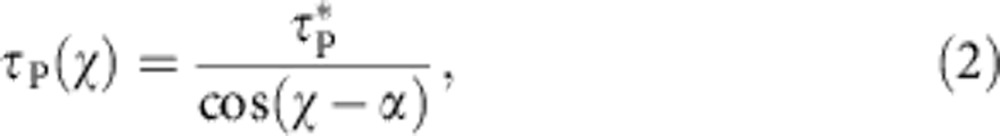


depending on two material-dependent parameters, 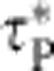 and *α*.

### Comparison with experiments

To test the accuracy of the above law, we wish to compare its predictions with experimental data. We will consider three metals, Nb, Mo and Fe, for which very low-temperature (4.2 K) measurements of the yield stress variations with crystal orientation are available and provide the best experimental estimates for the screw dislocation Peierls stress^12^. Reproducing Peierls stresses from atomistic calculations is notoriously difficult^29^,^30^,^31^ because of the need to account for zero-point vibrations that play an important role in dislocation glide at low temperatures^31^,^32^ and non-glide stresses that strongly affect the Peierls stress^14^,^16^,^17^,^33^. Both effects would be extremely computationally expensive to include. For this reason, and because we are interested here in the asymmetry of the Peierls stress rather than its magnitude, we have taken the values of the *α*-angles from our DFT zero-stress calculations in Table 1; however, we have chosen 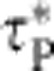 so as to reproduce the experimental value at *χ*=0°.

The predictions from equation (2) are compared with the experimental data in Fig. 2b. We obtain a qualitatively good agreement, especially considering the simplicity of equation (2). It is noteworthy that we recover that Nb has the strongest T/AT asymmetry, Mo is intermediate and *α*-Fe is almost symmetrical. This agreement shows that the T/AT asymmetry is a direct consequence of the deviation of the dislocation trajectory from a straight path, with larger deviations (more negative *α*-angles, or equivalently, saddle states closer to a [111] atomic column) leading to larger asymmetries. The fact that the yield stress is systematically lower in the T region than in the AT region^3^ is therefore directly connected to *α* being negative in all BCC metals. Discrepancies between the modified Schmid law and the experiments may arise from non-glide stresses that are present in the experiments but are not accounted for in equation (2).

So far, non-Schmid effects have been modelled using a phenomenological law based on a linear combination of the stresses that affect dislocation motion, which in the case of the T/AT asymmetry are the RSSs projected on two different planes of the 〈111〉 zone^14^. The choice of these planes is arbitrary as long as they are not parallel^33^,^34^. When the horizontal (1
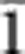
0) plane and the (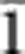
01) plane in the twinning region are chosen, the yield criterion is expressed as:


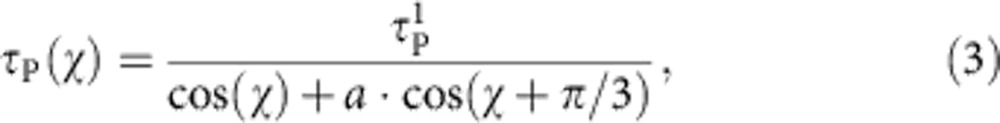


with 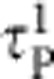 and *a*, two phenomenological parameters that may be fitted on experimental or numerical data. Expanding and reorganizing equations (2) and (3), we can show that 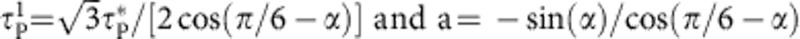. Equation (2) therefore provides a physical justification for the phenomenological law in equation (3). In particular, the relation above shows that *a* increases monotonically from 0 to 1 when *α* decreases from 0 (straight path) to −*π*/6 (path passing through the [111] atomic column). The parameter *a* is therefore directly related to the deviation of the dislocation trajectory from the straight (1
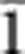
0) plane.

### DFT calculations under stress

To show that a quantitative agreement can be obtained with the modified Schmid law and avoid the difficulties mentioned above related to the comparison between experimental and atomistic data, we will now compare the predictions from equation (2) with DFT calculations of the Peierls stress. We consider metals from group VI, Mo and W, which slip along the {110} plane of maximum RSS at low temperatures^12^. Given the computational load related to such calculations, we studied three orientations: *χ*=0°, *χ*=−20° in the T region and *χ*=+20° in the AT region.

**Figure 3:**
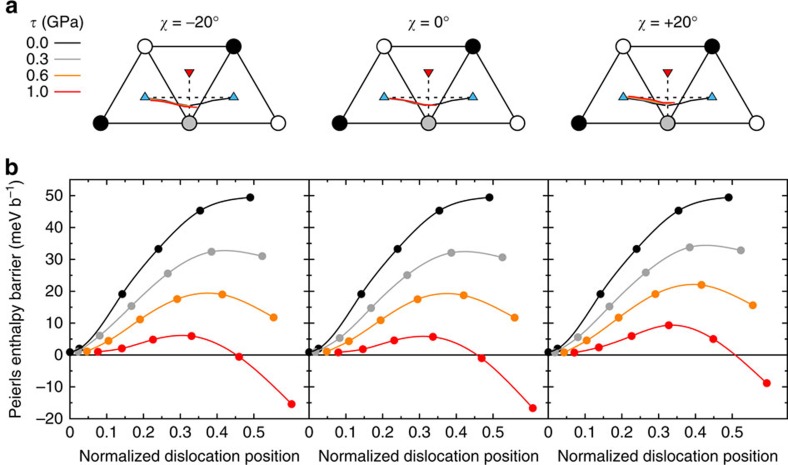
Dislocation trajectories and Peierls barriers under stress in Mo. Only the first half of the paths, which contain the enthalpy maxima, was calculated by DFT. In **a**, in the absence of applied stress, full trajectories are shown, owing to their symmetry (black lines). In **b**, dislocation positions are projected on the horizontal (1
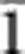
0) plane and normalized between 0 and 1 when the dislocation moves between the initial and final easy core configurations.

**Figure 4:**
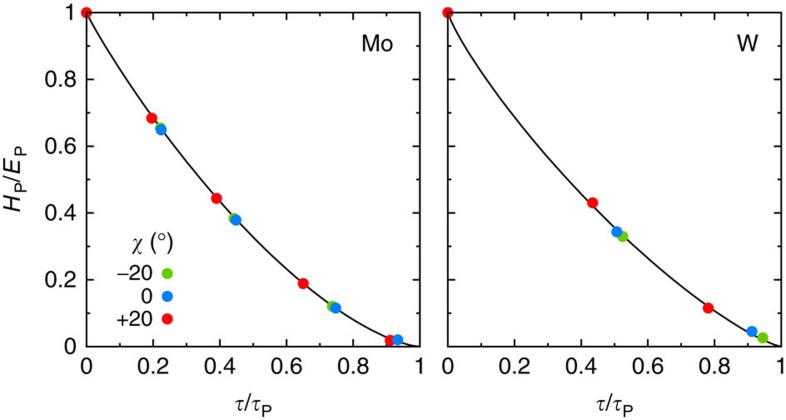
Normalized Peierls enthalpy variations with applied stress in Mo and W. The Peierls enthalpy *H*_P_ is normalized by the energy barrier, *E*_P_, calculated with DFT at zero applied stress. The applied stress, τ, is normalized by the Peierls stress, τ_P_ for each orientation. The symbols are the DFT data, the black lines the fits by Kocks-type power laws. We found that a single set of *p* and *q* parameters fitted all data for a given material ((*p*, *q*)=(0.94, 1.51) for Mo and (0.85, 1.27) for W).

To obtain Peierls stresses, we did not compute maximum derivatives on 2D Peierls potentials that are not accurate enough, but directly determined the applied stress that cancels the Peierls barrier. We first determined Peierls barriers under increasing simple shear stresses, *τ*, applied along the three orientations just mentioned (see Methods). To reduce the computational load, we computed only the first half of the paths where the activated state is located. The results are illustrated in Fig. 3 for Mo. As expected, the enthalpy barriers decrease with increasing applied stress. The corresponding dislocation trajectories do not depend on the applied stress, except for a small offset due to the displacement of the dislocation equilibrium position from the bottom of the Peierls valley. This will allow us in the following to use the values of *α* computed in the absence of applied stress (Table 1) to parametrize equation (2). From the barriers calculated at different applied stresses and orientations, we extracted the Peierls enthalpies, *H*_P_(*τ*,*χ*), the maximum of the Peierls barriers. The Peierls stresses, *τ*_P_(*χ*), were then obtained as the critical stresses at which the Peierls enthalpies disappeared. For this, we used a power law fit of Kocks type^35^, 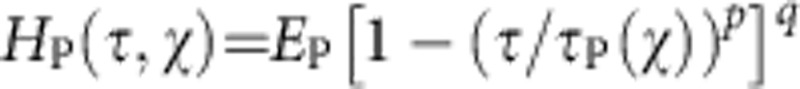. This law was originally proposed to model phenomenologically the stress dependence of the kink-pair formation enthalpy but, interestingly, we found here that the Peierls enthalpies follow the same trend. Our fits are therefore not to be used to predict dislocation kinetics, but only to extract Peierls stresses, *τ*_P_(*χ*). Moreover, as shown in Fig. 4, we performed calculations near the fitted *τ*_P_(*χ*), to ensure that the Peierls enthalpies vanished at these stresses.

**Figure 5:**
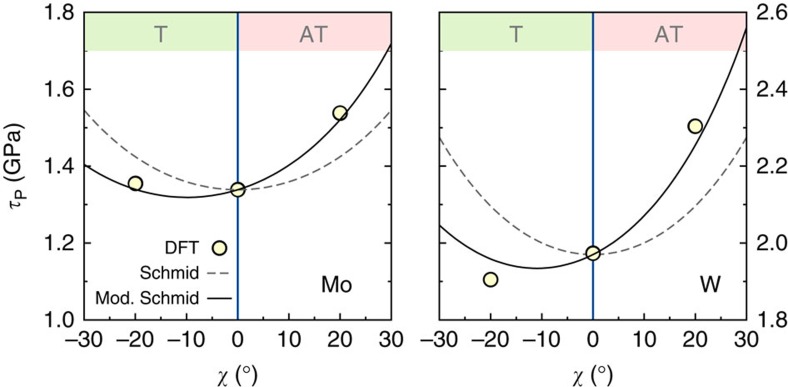
Peierls stress variations with crystal orientation in Mo and W. The Peierls stress variations calculated with DFT under stress (circles) are compared with the original Schmid law (dashed lines) and with the modified Schmid law from equation (2) (full lines).

From the results shown in Fig. 4, we obtained the Peierls stress variations with maximum RSS plane orientation in Mo and W displayed in Fig. 5 (circles). We find that W is more asymmetric than Mo, which is consistent with the fact that the *α*-angle in W is more negative than in Mo (see Table 1). In Fig. 5, we also reported the Peierls stress variations predicted by equation (2) (solid line). The values of 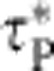 in this equation were set to reproduce the Peierls stresses obtained by DFT at *χ*=0°. We see that there is a very good agreement between the values obtained with DFT and the Peierls stresses predicted by the modified Schmid law. This confirms the accuracy of this law and the fact that the T/AT asymmetry is intimately related to the deviation of the dislocation trajectory from a straight line between equilibrium configurations.

Obviously, the present law is not limited to DFT calculations and can be applied to interatomic potentials. In particular, we know that the embedded atom method (EAM) potential developed by Mendelev *et al*.^23^ for Fe predicts a double-humped Peierls barrier that passes through the split core configuration, which is metastable with this potential^19^,^20^,^21^,^22^,^28^. As a result, we know without calculation that *α*=−*π*/6. In this case, equation (2) predicts the strongest T/AT asymmetry. This is confirmed by the Peierls stress calculations presented in ref. 10 and compared with equation (2) in Supplementary Fig. 2.

## Discussion

We showed that plastic anisotropy is linked to the 1/2〈111〉 screw dislocation trajectory and, more specifically, to its deviation from the (1
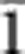
0) average glide plane. The deviation is itself related to the position of the saddle configuration systematically located in the T region. As the dislocation 2D Peierls potential is metal dependent, so is the deviation and thus the T/AT asymmetry. This origin of plastic anisotropy, evidenced by atomistic simulations, can be accounted for in a modified version of the Schmid law, where we showed that the component of the applied stress tensor that affects dislocation motion is the shear stress resolved on the actual inclined trajectory rather than the average (1
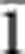
0) plane. The saddle point located in the T region thus reconciles the apparent paradox of a core energy higher for the split than for the hard core with simultaneously a resistance lower in the T region than in the AT region^19^,^20^,^28^.

With this new understanding of the T/AT asymmetry at 0 K, it will now be possible to model plastic anisotropy at finite temperatures. Line tension models, using the Peierls barrier as input parameter, have proven to faithfully describe the kink-pair mechanism operating at finite temperatures down to rather low stresses^26^,^36^,^37^. To investigate the T/AT asymmetry evolution with temperature, 2D Peierls potentials will need to be considered. Using phenomenological potentials, Edagawa *et al*.^24^,^25^ have shown that this approach reproduces the temperature dependence of the T/AT asymmetry observed experimentally in BCC transition metals. One should now employ atomistically determined 2D potentials^20^. We expect that the dislocation segments inside the nucleating kink pairs will follow a curved path similar to the straight dislocation, to minimize their core energy, confirming the importance of the dislocation trajectory on the T/AT asymmetry at finite temperatures.

It will also now be of prime importance to consider the other aspect of plastic anisotropy, that is, the effect of non-glide stresses. These stresses are resolved perpendicularly to the dislocation Burgers vector. They do not produce any force on the dislocation but still strongly affect its core structure, Peierls stress and mobility^9^,^15^. Phenomenologically, non-glide stresses have been incorporated in the linear combination of equation (3) (refs 16, 17, 38), but it remains to be evaluated how these stresses affect the dislocation trajectory and whether they may be incorporated in equation (2) as an additional dependence of the deviation angle. Such DFT calculations are extremely computationally expensive but would allow for a complete and physical picture of plastic anisotropy in BCC metals.

## Methods

### DFT calculations

The first-principles electronic structure calculations were performed within the DFT framework using the PWSCF plane-wave code^39^. The pseudopotentials are ultrasoft with semicore electrons for group V and VI elements, and without semicore electrons but with nonlinear core corrections for Fe. Residual forces after relaxation are smaller than 10^−2^ eV Å^−1^. The Hermite–Gauss scheme to broaden the electronic density of states was used with a smearing of 0.3 eV. The calculations in Fe are spin polarized and the Perdew–Burke–Ernzerhof generalized gradient approximation was used with a wave-function cutoff of 40 Ry and a shifted 1 × 2 × 16 *k*-point grid for all elements. No symmetry operator was applied apart from the inversion symmetry. The calculations were performed using a periodic array of dislocation dipoles with a quadrupolar arrangement, as in previous studies^19^,^20^,^40^,^41^, where we have carefully checked that we correctly obtain the properties of an isolated dislocation by correcting for the elastic interactions with the periodic images. In particular, we showed that the position of the saddle point does not depend on the cell size (see Fig. 15 in ref. 20).

### Calculations under stress

We applied pure shear stresses along the [111] axis:


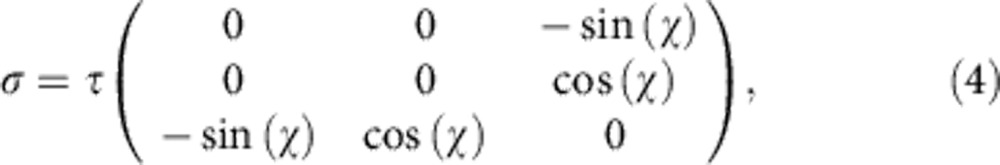


where *τ* is the stress resolved in the maximum RSS plane and *χ* is the angle between the maximum RSS plane and the (1
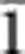
0) plane (see Fig. 1). Stresses were applied by straining the periodicity vectors of the simulation cell, with the applied strain *ɛ* related to the target stress by^41^:


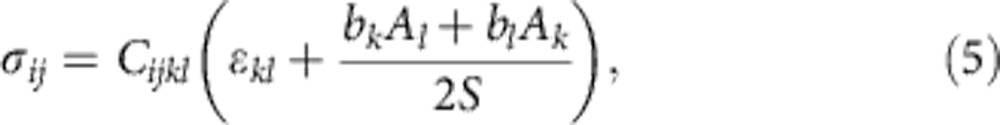


where *S* is the area of the simulation cell perpendicular to the dislocation lines and *C*_*ijkl*_ are the elastic constants computed from *ab initio*. For dislocations with a line direction along the *z* axis, the cut vector 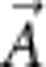 is given by *A*_*x*_=*y*_1_−*y*_2_ and *A*_*y*_=*x*_2_−*x*_1_, where (*x*_1_, *y*_1_) and (*x*_2_, *y*_2_) are the coordinates of the dislocations with Burgers vectors 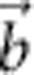 and −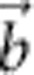, respectively.

### Peierls barriers

The Peierls barriers were calculated at fixed periodicity vectors by displacing one dislocation of the dipole, while the second dislocation remained immobile (see Supplementary Fig. 3). The initial paths were discretized linearly and the first half of each path was relaxed using the reaction coordinate method^42^, which is known to predict accurately minimum energy paths in the case of simple paths as considered here^19^. To avoid the reaction coordinate being maintained by the addition of small displacements spread over all the atoms of the simulation cell (instead of large displacements around the dislocation core as intended), the constraint was imposed only to the five atoms closest to the dislocation core. As the distance between dislocations varies along the paths, so does their elastic interaction energy. Once the dislocation positions were determined (see below), we withdrew this elastic contribution from the *ab initio* energy variation using anisotropic linear elasticity^20^,^41^,^43^ (see Supplementary Fig. 3 for a validation of this approach).

### Dislocation position

Because of the fixed periodicity vectors and of the varying distance between the two dislocations composing the dipole, a stress variation is observed in the simulations. One can deduce from this variation the dislocation positions^44^. Inverting the system in equation (5) for the 〈111〉 screw dislocation, the positions are given by:


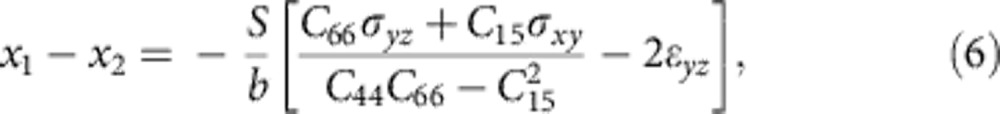



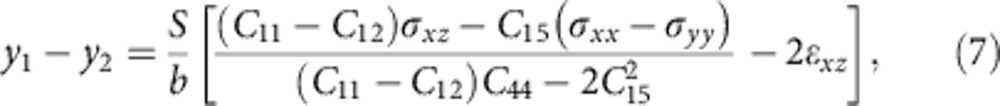


with the elastic constants written in Voigt notation in the dislocation frame of reference, that is, with *x*, *y* and *z* along the [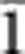

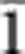
2], [1
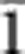
0] and [111] directions, respectively. Equations (6) and (7) provide the relative positions of the dislocations. To obtain absolute positions, we first consider that, when dislocations are at rest, that is, in their equilibrium initial and final positions, they are both displaced by the same distance from the bottom of their Peierls valleys but in opposite directions. We then assume that the position of the dislocation staying in the same Peierls valley varies linearly between the initial and final configurations, a reasonable assumption in view of the small displacement experienced by this dislocation compared with the gliding dislocation.

We checked that the predictions of the modified Schmid law do not rely on the definition used for the dislocation position by performing all the postprocessing mentionned above with a second definition for the dislocation position: the ‘cost function method' that consists in adjusting the atomic positions calculated with DFT to the atomic positions calculated for a compact screw dislocation within anisotropic elasticity by minimization of a cost function. This method is detailed in refs 19, 20. All the obtained data are given in Supplementary Figs 1 and 4 and in Supplementary Table 1. The values of *α* calculated with the cost function method follows the same trend as those calculated with the stress-based method and we find again a very good agreement between the DFT values and the predictions from the modified Schmid law, thus confirming the validity of the present approach.

### Data availability

The data that support the findings of this study are available from the corresponding author on request.

## Additional information

**How to cite this article:** Dezerald, L. *et al*. Plastic anisotropy and dislocation trajectory in BCC metals. *Nat. Commun.* 7:11695 doi: 10.1038/ncomms11695 (2016).

## Supplementary Material

Supplementary Information Supplementary Figures 1-4, Supplementary Table 1 and Supplementary References

## Figures and Tables

**Table 1:** Deviation angle and energy ratios from DFT calculations^20^ in the absence of applied stress in all BCC transition metals.

	V	Nb	Ta	Mo	W	Fe
*α* (°)	−10.6	−21.7	−7.1	−9.9	−10.9	−1.7
*E*_S_/*E*_H_	0.98	1.03	1.48	1.67	1.62	2.73

BCC, body-centred cubic; DFT, density functional theory.
