# Supplementary material for: Plastic anisotropy and dislocation trajectory in BCC metals
Source: Nat Commun. 2016 May 25;7:11695. doi: 10.1038/ncomms11695 (PMC4894953; doi:10.1038/ncomms11695)
Supplement: Supplementary Information — Supplementary Figures 1-4, Supplementary Table 1 and Supplementary References [file ncomms11695-s1.pdf]

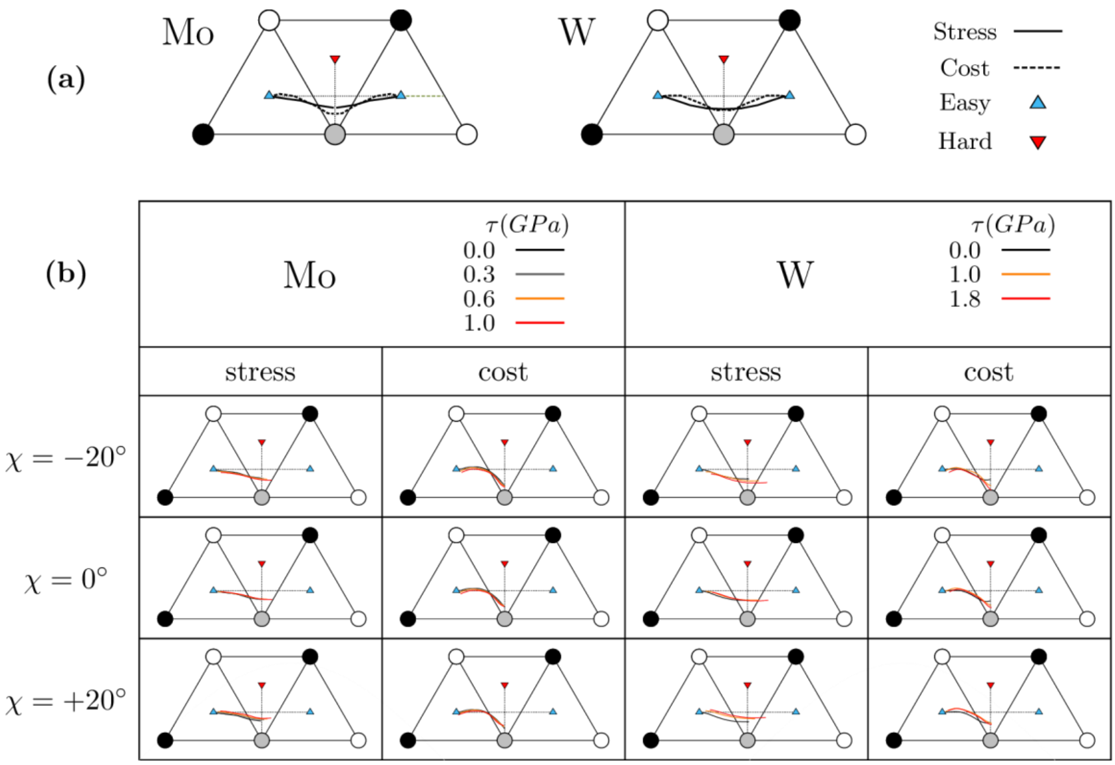

Supplementary Figure 1: Dislocation trajectories under applied stress. Comparison of the trajectories obtained with two methods: the cost function method and the stress-based method in absence (a) and in presence (b) of applied stress. In absence of stress, the trajectories were symmetrized to show the full paths, while under stress, such symmetrization is not possible and only the calculated half paths are shown.

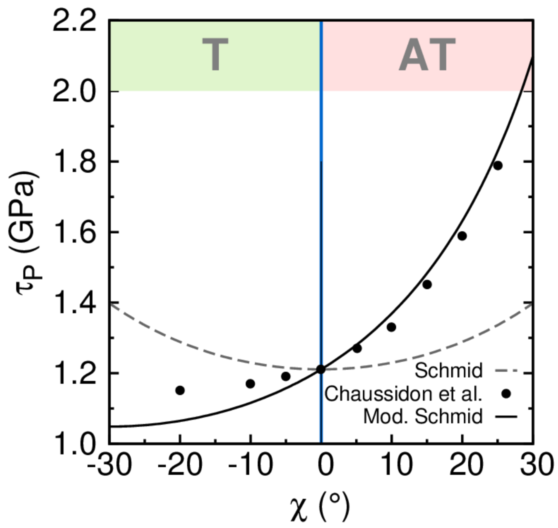

Supplementary Figure 2: Modified Schmid law applied to interatomic potentials. The predictions from the modified Schmid law (full line) is compared to the calculations from Ref. [2] (circles) and to the original Schmid law (dashed line). The interatomic potential is the Mendelev et al. Embedded Atom Method potential from Ref. [3].

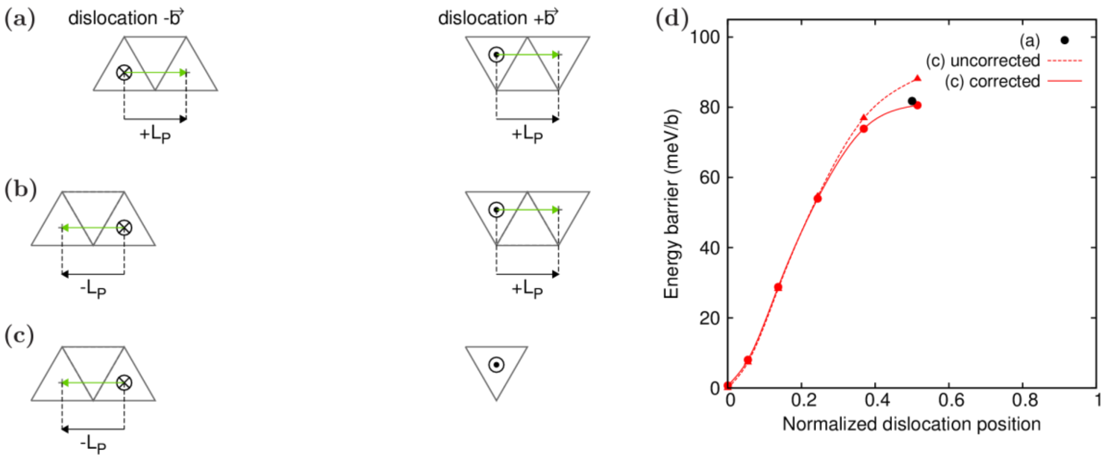

Supplementary Figure 3: Reaction coordinates to evaluate Peierls barriers. (a) Reference reaction coordinate, where both dislocations of the dipole move in the same direction. (b) Illustration of the displacement in opposite directions for dislocations under stress. (c) Reaction coordinate used in the present study, where only one dislocation is moved. (d) Peierls energies in tungsten for the reference (black) and present (red) reaction coordinates. In the latter case, the energy barrier is shown before (dashed line) and after (solid line) elastic corrections. Note that for the reference reaction coordinate, we show only the maximum of the Peierls energy, which by symmetry is halfway along the path, because the definition of the dislocation position requires that only one dislocation moves and can therefore not be applied with the reference reaction coordinate. The reference Peierls energy was shown to not require elastic correction in Ref. [1] since the elastic energy induced when moving the two dislocations simultaneously does not exceed 1 meV/b.

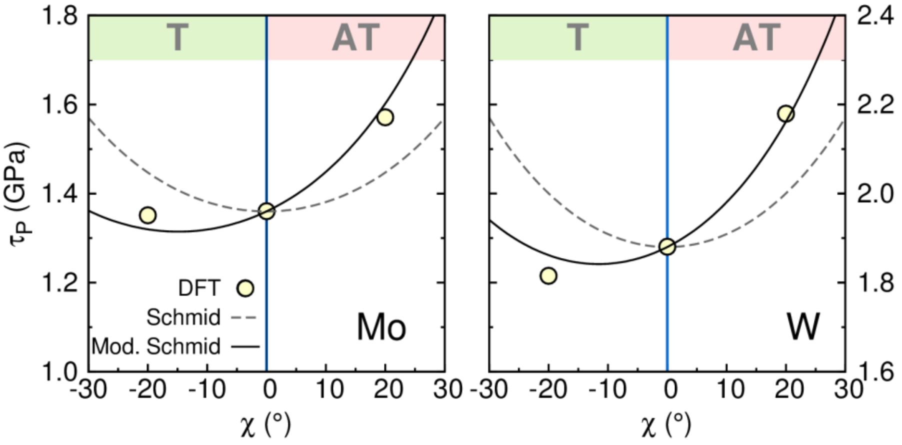

Supplementary Figure 4: Schmid law deviation obtained with the cost function method. The Peierls stress variations calculated with DFT under stress (circles) are compared to the original Schmid law (dashed lines) and to the modified Schmid law (full lines).

Supplementary Table 1: Deviation angle obtained from DFT calculations in absence of applied stress in all bcc transition metals evaluated with the cost function method.

	V	Nb	Ta	Mo	W	Fe
Cost function	-18.9	-24.7	-7.2	-14.9	-11.6	-5.3

Supplementary References

[1] Dezerald, L. *et al.* Ab initio modeling of the two-dimensional energy landscape of screw dislocations in bcc transition metals. *Phys. Rev. B* 89, 024104–13 (2014).

[2] Chaussidon, J., Fivel, M. & Rodney, D. The glide of screw dislocations in BCC Fe: Atomistic static and dynamic simulations. *Acta Mater.* 54, 3407–3416 (2006).

[3] Mendelev, M. I. *et al.* Development of new interatomic potentials appropriate for crystalline and liquid iron. *Philos. Mag.* 83, 3977–3994 (2003).
